# Imatinib pharmacokinetics and creatine kinase levels in chronic myeloid leukemia patients: implications for therapeutic response and monitoring

**DOI:** 10.1007/s00228-024-03675-9

**Published:** 2024-03-27

**Authors:** Mervat M. Omran, Amel B. Ibrahim, Raafat Abdelfattah, Samia A. Shouman, Marwa S. Hamza

**Affiliations:** 1 Pharmacology Unit, Cancer Biology Department, National Cancer Institute, Cairo University, Cairo, 11796 Egypt; 2 Department of Pharmacology, Faculty of Medicine, Zawia University, Zawia, Libya; 3 Medical Oncology Department, National Cancer Institute, Cairo University, Cairo, Egypt; 4 Clinical Pharmacy Practice Department, Faculty of Pharmacy, The British University in Egypt, El-Sherouk City, Cairo, Egypt

**Keywords:** Imatinib, Chronic myeloid leukemia, CK levels, CK-MB, Pharmacokinetics

## Abstract

**Background:**

Imatinib treatment for certain cancers can lead to elevated creatine kinase (CK) levels, potentially indicating muscle injury, and ongoing research aims to understand the correlation between imatinib levels and creatine kinase to assess its impact on treatment response.

**Methods:**

This single-center observational study involved 76 chronic myeloid leukemia (CML) patients receiving imatinib treatment, focusing on evaluating drug and metabolite levels using liquid chromatography–mass spectrometry (LC–MS-MS) instrumentation. Serum CK and creatine kinase-MB (CK-MB) levels were assessed using Colorimetric kits.

**Results:**

CK and CK-MB levels were measured, CK showed a median value of 211.5 IU/l and CK-MB showed a median value of 4.4 IU/l. Comparing low and high CK groups, significant differences were found in peak and trough plasma concentrations of imatinib and its metabolites. Correlations between CK levels and pharmacokinetic parameters were explored, with notable associations identified. Binary logistic regression revealed predictors influencing the therapeutic response to imatinib and categorized expected CK levels into high or low, with peak levels of imatinib emerging as a significant predictor for CK level categorization.

**Conclusion:**

The study highlights the link between imatinib’s pharmacokinetics and elevated CK levels, indicating a possible correlation between specific metabolites and improved treatment response. Individualized monitoring of CK levels and imatinib pharmacokinetics could enhance care for CML patients.

**Supplementary Information:**

The online version contains supplementary material available at 10.1007/s00228-024-03675-9.

## Background

Imatinib is commonly utilized as the first-line treatment for chronic myeloid leukemia (CML) due to its low risk and favorable side effect profile [1]. A previous study reports that some patients with gastrointestinal stromal tumors (GIST) or CML and treated with imatinib showed elevated serum creatine kinase (CK), indicating muscle injury, muscular dystrophy, and myocardial infarction [2, 3]. A significant number of CML patients using tyrosine kinase inhibitors (TKIs) report muscle complaints [4] and show increased CK levels, particularly in those treated with TKIs for over 6 months [5]. Rhabdomyolysis has been reported in two instances involving imatinib use [3, 6]. Gordon et al. described a case where 20 out of 25 imatinib-treated patients presented with increased CK levels [3]. Research has established a correlation between Imatinib dosage (400 mg vs. higher dosages) and elevated CK levels in CML patients [2]. Despite the frequent occurrence of elevated CK in individuals treated with imatinib, there is no evidence of myopathy during the follow-up period [2].

Furthermore, the absence of elevated CK-MB levels align with evidence supporting the cardiac safety of imatinib [7–9]. Testing of CK-MB levels in 6 patients with elevated total CK levels revealed normal values, indicating that the high CK levels in these patients did not originate from the myocardium [2]. Studies on rats have suggested that imatinib, whether used alone or in combination with desoxycorticosterone acetate, can lead to tissue damage, as evidenced by increased CK-MB levels, with these effects potentially associated with oxidative stress [10, 11]. The incidence of musculoskeletal issues in imatinib-treated patients surpasses the prevalence of CK elevation, yet the correlation between musculoskeletal symptoms and CK levels remains poorly elucidated [12].

Imatinib demonstrates high oral bioavailability and a consistent dose-exposure relationship, with a terminal half-life of approximately 20 h [13], but patient variability in drug disposition can affect treatment outcomes [14]. The active metabolite N-desmethyl imatinib has a lower cytotoxicity compared to the parent drug and accounts for about 20% of its plasma level [15], while two other N-oxide metabolites have been detected in urine shortly after dosing [16]. Some pharmacokinetic studies have shown a correlation between low plasma exposure and slower objective response rates, resistance development, or disease progression, this is not always the case [17]. Also, previous studies suggest that patients are more likely to have good treatment responses or improved response rates when administered imatinib at the appropriate trough concentration. Plasma levels of imatinib are shown to be higher in patients who obtain a complete cytogenetic response (CCR) compared to those who do not, and this trend is also seen in individuals with a major molecular response (MMR) [18, 19]. In addition, higher imatinib plasma levels may contribute to the swift and effective management of illness observed in patients with elevated CK possibly reflecting higher drug levels [12], but it is uncertain whether individuals with elevated CK experience improved clinical outcomes due to these heightened responses [20]. Previous research has indicated that over 70% of CML patients undergoing first-line TKI treatment continue to manifest elevated CK levels during follow-up, revealing a strong association between CK elevation during TKI therapy and enhanced overall and event-free survival in newly diagnosed chronic phase CML patients [20]. Another study showed a significant correlation between substantial cytogenetic response and prolonged CK elevation in CML patients receiving TKI treatment [21]. Moreover, patients with elevated CK demonstrate improved overall-free survival, suggesting better disease management possibly due to higher medication levels and fewer treatment-related events (including treatment failures or deaths) [20]. While the overall number of deaths was minimal, no significant alterations in causes of death related to CK levels were observed [22, 23]. Consequently, CK elevation is generally regarded as asymptomatic in the vast majority of patients [24, 25]. Elevated CK levels are linked to major cytogenetic remission, possibly reflecting increased drug levels, as confirmed by a previous study [21].

In a prospective study, they reported elevated CK levels, with no discernible link between these heightened CK levels and musculoskeletal complaints. This may be attributed to the fact that over 90% of these patients were on low doses of imatinib [26]. Another prospective trial revealed that 80% of patients exhibited a persistent rise in CK (> 50% above baseline) 6 months after initiating imatinib, and over half of CML patients treated with imatinib experienced CK elevation within the first year of follow-up [2, 12]. Further investigation into pharmacokinetics is necessary to determine whether individuals with elevated CK have heightened exposure to medication levels associated with improved treatment outcomes [7, 26]. Therefore, the objective of this study is to explore the correlation between plasma concentrations of imatinib and plasma levels of CK and CK-MB and to assess how this relationship impacts the therapeutic response to imatinib treatment.

## Patients and methods

### Study design, patients, and data sources

The present study is a prospective single-center observational study. Patients receiving imatinib as a first-line treatment for chronic myeloid leukemia (CML) were included in the study. Participation from 76 patients was recorded in this study, which took place at the Hematological Outpatient Clinic of the National Cancer Institute, Cairo University. The criteria for inclusion in the study were a new diagnosis of CML, an age of 18 years or older, and at least 30 days of imatinib treatment (to achieve a steady state). The determinations for this study included plasma levels of peak and trough imatinib and its metabolites, as well as the plasma concentration of creatine kinase (CK). Prior to its commencement, the study protocol was granted approval by the Institutional Review Board (IRB00004025) of the National Cancer Institute of Cairo University, Egypt. In adherence to the guidelines of the Declaration of Helsinki, written informed consent was secured from all participants.

### Response assessment

According to the European LeukemiaNet guidelines (ELN), the patient’s response to treatment can be monitored through cytogenetic (karyotype and/or FISH) and molecular (detection of BCR-ABL1 mRNA metaphases) assessments. These evaluations aid in identifying optimal and suboptimal responses as well as treatment failure based on molecular and cytogenetic responses throughout the treatment process [27].

### Blood sampling and detection of imatinib and its metabolites

#### Samples preparation

Blood samples were obtained in EDTA-containing tubes immediately before drug administration (trough sample) and 2 h after Imatinib administration (peak sample) as previously shown [28]. The plasma was separated through centrifugation at 2500 × g for 10 min, where 400 µl of plasma was transferred to a glass tube and combined with 40 µl of the internal standard (IS) stock solution (2 ug/ml), followed by the addition of 1200 µl of methanol. The mixture was vortexed and then centrifuged at 10,000 × g at 4 °C for 10 min. The resulting clear supernatant was transferred to HPLC autosampler vials, and 10 µl was injected into the LC/MS/MS system [29].

### LC–MS-MS instrumentation and operating conditions

The LC–MS-MS system utilized an ABSCIEX Q TRAP 3200 mass spectrometer (ABSCIEX, Germany) with an electrospray ionization (ESI) interface, linked to an Agilent 1200 HPLC system (Agilent Technologies, CA, USA) featuring a quaternary gradient pump (Agilent 1260 infinity) and an autosampler (Agilent 1260 infinity). Data acquisition was conducted using analyst 4.0 software (ABSCIEX). Separation was carried out employing an Agilent pro shell EC, C18 (5 µm, 50 × 4.6 mm) reversed-phase analytical column (Agilent, CA, USA) as previously described by Omran et al. (2020). The mobile phase, consisting of 0.1% formic acid in methanol/water (55:45, v/v), was delivered at a flow rate of 700 µl/min, with a total run time of 6 min. The mass spectrometer operated in positive ESI mode at a temperature of 350 °C. Calculations were performed using the Multiquant software program. Quantification utilized multiple reaction monitoring (MRM) with the following ion transitions: m/z 494:394, 480:394, 510:217, and 297:110 for imatinib, N-des-methyl imatinib, pyridine-N-oxide imatinib, and palonosetron (IS), respectively.

### Calibration curves

The stock solutions of imatinib, N-des-methyl imatinib, pyridine-N-oxide imatinib, and palonosetron as internal standard (IS) were created by dissolving 1 mg of each drug in 1 ml of methanol/water (50:50). Serial dilutions were subsequently prepared, spanning concentrations from (4.8–5000) ng/ml for imatinib, (2.7–700) ng/ml for N-des-methyl imatinib, and (5.4–700) ng/ml for pyridine-N-oxide imatinib in drug-free plasma, and then spiked with the known concentration of the IS (Supplement S1-S3). Pharmacokinetics parameters were calculated as shown in the supplement file.

### Detection of human CK and CK-MB levels

Serum CK and CK-MB levels were quantified using Colorimetric kits (Spectrum, China) according to the manufacturer’s protocol.

### Statistical analysis

The determination of normality of data distribution was conducted using Shapiro–Wilk and Kolmogorov–Smirnov tests. We considered data to deviate from normality if the *p*-value was less than 0.05. Quantitative variables were presented using means and standard deviations or medians and interquartile ranges, while categorical variables were expressed as percentages. *T*-tests were utilized to analyze differences between two groups for normally distributed data. Conversely, the Mann–Whitney *U* test was applied for continuous data lacking a normal distribution. Statistical differences in categorical variables between groups were assessed using the chi-square test or Fisher’s exact test, as appropriate. The measure of strength of association between response and the predictors was reported using odds ratios (ORs). A similar analysis was performed for the secondary outcome of the level of creatine kinase. The significance threshold was 0.05 and testing was two-sided. Statistical analyses were conducted using SPSS.

## Results

### Patient demographics, medical conditions, and treatment response

The present study involved 76 patients who were treated by imatinib dose of 400 mg/ day. The mean age of the participants is 39.7 years. The gender distribution shows that 36 patients (47.3%) are male, while 40 (52.7%) were female. Medical conditions include 7 participants (9.2%) with hypertension and 5 (6.6%) with diabetes. The study also evaluates the response to treatment, with 46 individuals (60.5%) showing a good response and 30 (39.5%) showing a bad response. Laboratory test results include parameters such as hemoglobin levels, white blood cell count, platelet count, aspartate aminotransferase (AST), alanine aminotransferase (ALT), creatinine, and urea. Focusing on CK and CK-MB levels, their levels were measured in the study, with CK-MB having a median value of 4.4 IU/l (0.4 to 28.68 IU/l), while CK had a median value of 211.5 IU/l (50.19 to 393.43 IU/l) (Table 1).

**Table 1:** Demographic and clinical characteristics of study participants categorized by CK levels

	Overall (*n* = 76)	Low CK (*n* = 32)	High CK (n = 44)	*p*-value
Age (years) mean ± SD	39.7 ± 9.7	41.5 ± 9.5	38 ± 8.8	0.83
Gender				**0.024**
Male	36 (47.3%)	20 (55.6%)	16 (44.4%)	
Female	40 (52.7%)	12 (30%)	28 (70%)	
Hypertension	7 (9.2%)	2 (28.6%)	5 (71.4%)	0.45
Diabetes	5 (6.6%)	1 (20%)	4 (80%)	0.37
Response				0.51
Good response	46 (60.5%)	18 (39%)	28 (71%)	
Bad response	30 (39.5%)	14 (46.7%)	16 (53.3%)	
Hg (g/dl) mean ± SD	11.6 ± 2	11.5 ± 2	11.8 ± 2	0.52
WBC (/mm 3) median (range)	6 (2.5–185)	6.3 (2.9–185)	5.5 (2.5–7.8)	0.06
Platelet (/mm 3) median (range)	197.5 (14–934)	214 (14–554)	176 (40–934)	0.2
AST (IU) median (range)	22 (12.2–115.4)	2.51 (14–115.4)	22 (12.2–44)	0.6
ALT (IU) median (range)	17.6 (7.4–117)	18 (8.7–117)	18 (7.4–72.3)	0.6
Creatinine (mg/dl) mean ± SD	0.88 ± 0.19	0.9 ± 0.2	0.89 ± 0.16	0.2
Urea (mg/dl) mean ± SD	24.3 ± 6.7	26.3 ± 7.6	22 ± 6.2	0.7
CK-MB	4.4 (0.4–28.68)	4.6 (0.4–26.83)	3.8 (0.41–28.68)	0.5

Data bold indicate p value is significant

The entire study sample was categorized into two groups based on their CK levels, as per the guidelines provided in the kit manual. The standard CK range for men was established as 24–204 U/l and for women it was set at 24–173 U/l. Consequently, any male patient with a CK level exceeding 204 U/l and any female patient with a CK level surpassing 173 U/l were classified into the high CK level group. Conversely, any male patient with a CK level below 204 U/l and any female patient with a CK level below 173 U/l were included in the low CK level group. There was no significant difference observed between the low and high CK groups regarding the age. Although, in terms of gender, there are slightly more females (52.7%) than males (47.3%). The blood test results show no significant differences observed between the low and high CK groups regarding AST, ALT, creatinine, and urea levels (Table 1).

### Pharmacokinetic parameters and CK level groups

**Fig. 1:**
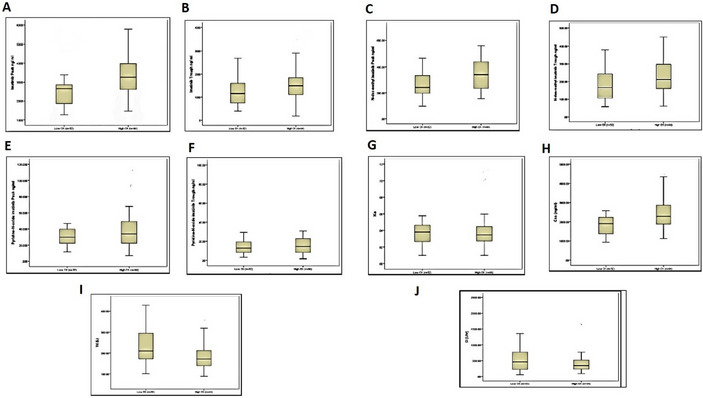
Pharmacokinetics characteristics of study participants categorized by CK levels: **A** peak imatinib plasma level (ng/ml), **B** trough imatinib plasma level (ng/ml), **C** peak N-des-methyl imatinib plasma level (ng/ml), **D** trough N-des-methyl imatinib plasma level (ng/ml), **E** peak pyridine-N-oxide imatinib plasma level (ng/ml), **F** trough pyridine-N-oxide imatinib plasma level (ng/ml), **G** Ke, **H** Css (ng/ml), **I** Vd (L), **J** Cl (l/h)

Figure 1 presents the pharmacokinetic parameters of imatinib and its metabolites, N-des-methyl imatinib, and pyridine-N-oxide imatinib, in patients classified into low and high CK level groups. Imatinib’s peak plasma concentrations demonstrated a significant difference between the low CK group, which exhibited a mean of 2665 ng/ml, and the high CK group, with a mean of 3166.5 ng/ml (*p*-value 0.004). The trough plasma concentrations of imatinib also varied significantly between the two groups, with the low CK group displaying a mean of 1169.5 ng/ml and the high CK group showing a mean of 1422 ng/ml (*p*-value 0.027). In terms of N-des-methyl imatinib, the peak plasma concentrations were significantly different, with the low CK group demonstrating a mean of 267 ng/ml and the high CK group showing a mean of 337.6 ng/ml (*p*-value 0.03). However, the trough levels did not reflect a significant difference between the two groups. The table also details other pharmacokinetic parameters, including clearance (Cl), volume of distribution (Vd), and elimination rate constant (Ke). The Cl values for imatinib did not differ significantly between the low and high CK groups. However, the volume of distribution did show a significant difference, with the low CK group presenting a mean Vd of 205 L and the high CK group a mean Vd of 178 L (p-value 0.004), as displayed in Fig. 1.

In our analysis of the correlation between CK and CK-MB levels and all pharmacokinetic parameters, we found no significant correlation between CK-MB plasma levels and all the pharmacokinetic parameters. However, the most pronounced positive correlation coefficient, 0.27, was observed between high levels of CK and peak plasma levels of imatinib (*p*-value: 0.02). Additionally, a positive correlation emerged between the CK levels and the steady state of imatinib, while a negative correlation was found between CK levels and the volume of distribution of imatinib (correlation coefficients of 0.26 and − 0.32 respectively, with corresponding *p*-values of 0.02 and 0.005).

### Predictors of therapeutic response and CK level classification

Utilizing binary logistic regression, the study was designed to discern predictors that could influence the therapeutic response to imatinib and to classify expected CK levels into either high or low categories. As detailed in Table 2, four variables were scrutinized: imatinib peak level, imatinib trough level, CK level, and pyridine-N-oxide trough levels. Statistically significant associations were identified between a favorable response to imatinib and several variables. In the analysis of therapeutic response to imatinib, our findings indicate a direct correlation with its peak plasma concentration. Specifically, for each increment of 1 ng/ml in imatinib peak concentration, we observed a corresponding increase in the probability of a positive therapeutic response, with an approximate factor of 1 (95% confidence interval [CI], 0.998 to 1.000). In parallel, an elevation in creatine kinase (CK) levels by 1 IU/l was associated with an analogous increase in the likelihood of a favorable response to imatinib treatment (95% CI, 1.002 to 1.015). Furthermore, binary logistic regression was utilized to elucidate the determinants of creatine kinase level classification as either high or low, as presented in Table 3. This analysis prominently underscores the peak concentration of imatinib as a significant predictor. Notably, there was a statistically significant relationship between the classification into the high CK level category and increased peak imatinib concentrations. Specifically, for each 1 ng/ml rise in the peak concentration of imatinib, there was a commensurate increase in the probability of classification into the high CK level group by a factor of approximately 1 (95% CI, 1.000 to 1.001).

**Table 2:** Binary logistic regression analysis of key variables influencing favorable response to imatinib treatment

	*B*	Sig	Exp(B)	95% C.I. for Exp(B)	
				Lower	Upper
Imatinib peak plasma level (ng/ml)	− 0.001	**0.039**	0.999	0.998	1.000
Imatinib trough plasma level (ng/ml)	0.001	0.113	1.001	1.000	1.003
CK (IU/l)	0.009	**0.011**	1.009	1.002	1.015
Pyridine-N-oxide imatinib trough plasma level (ng/ml)	0.067	0.081	1.069	0.992	1.153

Data bold indicate p value is significant

**Table 3:** Binary logistic regression analysis of imatinib peak levels as a predictor for CK level categorization

	*B*	Sig	Exp(B)	95% C.I. for Exp(B)	
				Lower	Upper
Imatinib peak plasma level (ng/ml)	0.001	0.009	1.001	1.000	1.001

Finally, the study delineates two groups based on imatinib trough concentrations, distinguishing those with therapeutic levels (> 1000 ng/ml) from those with levels considered subtherapeutic. Statistical analysis using the chi-square test demonstrates a significant association between the concentration of imatinib and CK levels, with a *p*-value of 0.028. This indicates a less than 3% likelihood that this observed correlation is due to chance, underscoring a consequential relationship between blood levels of imatinib and the high or low categorization of CK levels.

## Discussion

Imatinib is a tyrosine kinase inhibitor used to treat chronic myeloid leukemia (CML) and some forms of gastrointestinal stromal tumors (GISTs). It inhibits the enzymes necessary for the growth and survival of cancer cells. A large proportion of patients with CML or GIST experience CK rises when using Imatinib. Imatinib has been shown to have a deleterious effect on muscular tissue [2, 3, 6, 30]. It is unknown how imatinib could raise CK levels and myalgia is a typical imatinib side effect, especially when the drug is first administered [31]. It was reported that hypophosphatemia and hypocalcemia are two examples of electrolyte disorders that can be brought on by imatinib [32] and hypophosphatemia is associated with CK increase [2]. Since muscle tissue expresses the tyrosine kinases platelet-derived growth factor receptor (PDGFR) and c-Abl [33, 34], it is expected that inhibition of these enzymes by imatinib contributes to this damage [3].

CML is not usually connected with CK levels, but certain drugs, such as Imatinib, can impact them. Imatinib’s effect on CK levels may be affected by medication pharmacokinetics, patient characteristics, and other medical conditions. Imatinib duration and dosage may also affect CK levels.

We first compared CML patients’ Imatinib plasma levels to their CK and CK-MB plasma levels, we discovered that CK levels, but not CK-MB levels, were significantly associated with Imatinib pharmacokinetics, provides a hint that the source of elevated CK could be from tissues other than the heart, possibly skeletal muscle. This is further supported by the observed myalgia in some patients, a known side effect of Imatinib. Interestingly, we reported that greater CK levels were linked with higher Imatinib peak, trough, and N-des-methyl peak levels.

Moreover, this study examined various factors to understand their potential influence on the therapeutic response to Imatinib. These factors included the peak and trough levels of Imatinib, CK plasma level, and the trough levels of Pyridine-N-oxide, a metabolite of Imatinib. A significant association was found between the peak concentration of Imatinib and a positive response to the medication. The current investigation reveals a significant correlation between CK levels and the therapeutic efficacy of Imatinib. This association was further elucidated by stratifying patients into two cohorts predicated on their Imatinib trough concentrations, thereby differentiating between those with therapeutic levels (exceeding 1000 ng/ml) and those with subtherapeutic levels. There is a link between the concentration of Imatinib in the bloodstream and the classification of CK levels as either high or low. Consequently, it can be inferred that patients lacking therapeutically targeted Imatinib levels are not susceptible to high CK levels. Treatment outcomes may be influenced by Imatinib pharmacokinetics and the patient’s physiological condition, as evidenced by CK levels. It is important to note that Imatinib is not the only factor affecting CK levels; variables such as exercise, muscle damage, and other illnesses can also play a role [35].

In addition, the results showed that the response of CML patients to Imatinib is influenced by several factors, including CK levels, pyridine-N-oxide Imatinib levels, and trough imatinib levels. These concentrations significantly affect the efficacy and safety of imatinib. Remarkably, CML patients exhibit improved therapeutic responses when trough and peak imatinib levels are elevated. Enhanced drug exposure in CML patients correlates with increased frequencies of complete cytogenetic response (CCyR) and major molecular response (MMR). In addition, the therapeutic outcomes were enhanced when plasma levels of the pyridine-N-oxide imatinib metabolite were elevated.

Although it was found before that CK level is greater in men than in women [36] but in our study, females have higher levels of CK than males that may be explained by the nonpathological factors that affect CK levels such as age, exercise, time of day, and estrogen status of the female [37]. It has been widely documented in the literature that there are gender variations in muscle disruption and repair mechanisms. Research conducted on female animals has revealed reduced basal levels of CK and a diminished CK response to physical activity [38, 39]. This implies that CK levels can be greatly influenced by nonpathological variables like exercise, which may cause females to have higher amounts of CK even though their baseline levels are lower [40]. There is little to no difference in the response of males and females to eccentric exercise-induced muscle damage, and many of the findings that suggest women have an attenuated response to muscle damaging exercise may be more specifically applicable to aerobic exercise [41]. This opinion is corroborated by a review by Clarkson and Hubal (2002), who find that there are not many gender differences and suggest that women may be more susceptible to muscle disruption than men [42].

High CK levels were also linked to the steady-state concentration and volume of distribution of Imatinib. Higher CK levels in patients on Imatinib may be the cause of the increased frequency of myalgia [18], which was found to occur after 1 month of treatment at the normal 400 mg/day dose. The pharmacokinetic (PK) variability, which has been reported to be around 60% in the steady-state trough concentrations of Imatinib in patients with GISTs and 71% in CML, is a potential cause of interpatient heterogeneity in treatment response [43, 44]. Plasma trough levels (Cmin) in individuals receiving the same dosage—typically 400 mg/day of Imatinib—can vary widely due to PK variability, which is primarily caused by physiological, clinical, genetic, demographic, and environmental variables [45]. Approximately half of the patients with GISTs who had Imatinib experienced myelosuppression and periocular edema [46]. Since fixed dosages of medication are typically given for an extended length of time, therapeutic drug monitoring (TDM) has been suggested for use in this treatment [47].

The current study does have certain caveats. We did not have a baseline CK value, so it is possible that some of the patients who experienced a rise in CK levels in this study already had aberrant CK levels prior to starting Imatinib. Increases in asymptomatic CK level can be linked to factors including male gender and younger patient age. In conclusion, the results of the present study shed light on how imatinib affects CK levels in CML patients. The main results point to a possible effect of Imatinib on skeletal muscle instead of cardiac muscle and that shows the effect of Imatinib pharmacokinetics on the rise in CK levels. The present study also suggests that elevated peak and trough Imatinib levels may be linked to better therapeutic response. Ultimately, the research results could assist clinicians by offering indicators for proactive patient monitoring, directing personalized treatment modifications, and potentially enhancing patient outcomes with customized therapeutic approaches. Healthcare providers can integrate these findings into their clinical practice by establishing guidelines for monitoring creatine kinase levels and imatinib pharmacokinetics in chronic myeloid leukemia patients, taking into account variables like age and gender in risk evaluations.

### Supplementary Information

Below is the link to the electronic supplementary material.

Supplementary file1 (DOCX 54.8 KB)

## Data Availability

Raw data were generated at the National Cancer Institute. Derived data supporting the findings of this study are available from the corresponding author on request. The data are not publicly available due to containing information that could compromise participant’s ethical approval and consent to participate.
