# Supplementary material for: Imatinib pharmacokinetics and creatine kinase levels in chronic myeloid leukemia patients: implications for therapeutic response and monitoring
Source: Eur J Clin Pharmacol. 2024 Mar 27;80(7):1061–8. doi: 10.1007/s00228-024-03675-9 (PMC11156749; doi:10.1007/s00228-024-03675-9)
Supplement: Supplementary file 1 — Supplementary file1 (DOCX 54.8 KB) [file 228_2024_3675_MOESM1_ESM.docx]

- **Pharmacokinetic Data Analyses**

The imatinib pharmacokinetic was described by one compartment model for oral drugs.

- K_e_= ln (P/T)/22
- Elimination half-lives (T_1/2_) were calculated from the slopes of the exponential terms (0.693/k_e_).
- The peak (P), trough (T) and steady state (C_ss_) concentration was generated from the experimental data. C_ss_ = (P_+_ T)/2
- Volume of distribution at steady-state (V_D_) was calculated by the following equation using C_ss_ as the blood plasma concentration :


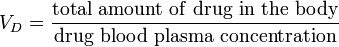


- Plasma clearance (Cl) = V_D_ K_e._
- Calibration curves were linear for each analyte (r^2^ ≥0.99) for IM, N-des-methyl imatinib and Pyridine-N-oxide imatinib.

**
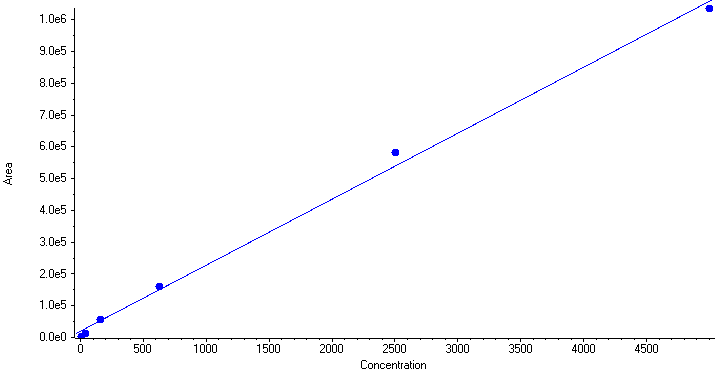
**

**y = 207.68739 x + 20115.45302 (r = 0.99820)**

**S1:** Calibration curves of Imatinib Mesylate (4.8-5000 ng/ml)**.**

**
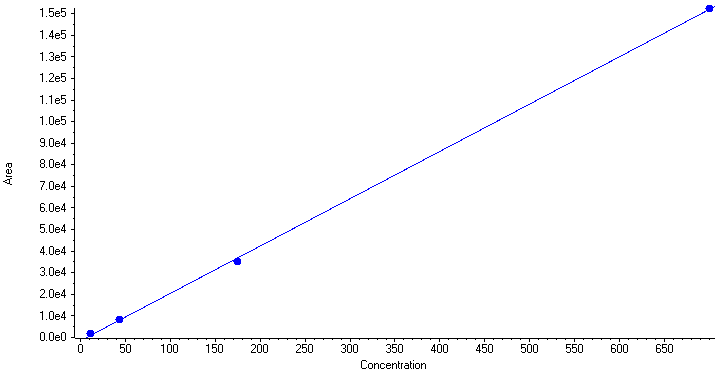
**

**y = 219.33761 x + -1479.94066 (r = 0.99987)**

**S2.** Calibration curve of N-des-methyl imatinib (5-700 ng/ml).

**
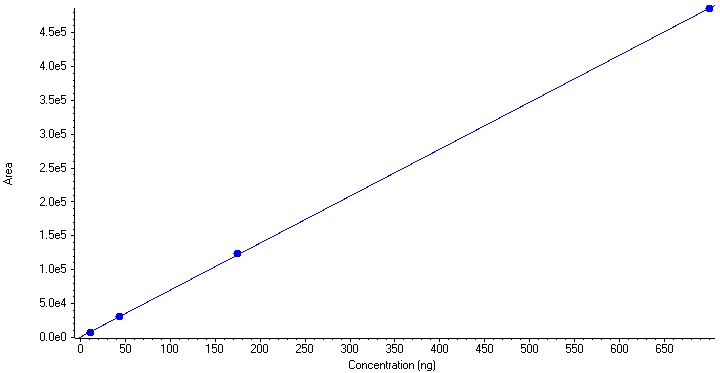
**

**y = 693.38025 x + 715.15085 (r = 0.99998)**

**S3.** Calibration curve of pyridine-N-oxide (5.4-700 ng/ml).
